# Forensic application of epidermal expression of HSP27 and HSP70 for the determination of wound vitality in human compressed neck skin

**DOI:** 10.1038/s41598-023-33799-4

**Published:** 2023-04-24

**Authors:** Siying Zhang, Yuko Ishida, Akiko Ishigami, Mizuho Nosaka, Yumi Kuninaka, Haruki Yasuda, Ayumi Kofuna, Jumpei Matsuki, Miyu Osako, Wei Zhang, Akihiko Kimura, Fukumi Furukawa, Toshikazu Kondo

**Affiliations:** Department of Forensic Medicine, Wakayama Medical University, 811-1 Kimiidera, Wakayama, 641-8509 Japan

**Keywords:** Medical research, Diagnostic markers

## Abstract

Estimating the age and vitality of human skin wounds is essential in forensic practice, and the use of immunohistochemical parameters in this regard remains a challenge. Heat shock proteins (HSPs) are evolutionarily conserved universal proteins that protect biological systems from various types of stress. However, its importance in forensic pathology for determining wound activation in neck compression skin remains unclear. The expression of HSP27 and HSP70 in neck skin samples was immunohistochemically examined to understand its forensic applicability in determining wound vitality. Skin samples were obtained from 45 cases of neck compression (hanging, 32 cases; strangulation, 10 cases; manual strangulation, 2 cases; other, 1 case) during forensic autopsies; intact skin from the same individual was used as a control. HSP27 expression was detected in 17.4% of keratinocytes in the intact skin samples. In the compressed region, the frequency of HSP27 expression in keratinocytes was 75.8%, which was significantly higher than that in intact skin. Similarly, HSP70 expression was 24.8% in intact skin samples and 81.9% in compressed skin samples, significantly higher in compressed skin than in intact skin samples. This increase in case compression cases may be due to the cell defence role of HSPs. From a forensic pathology perspective, the immunohistochemical examination of HSP27 and HSP70 expression in neck skin could be considered a valuable marker for diagnosing traces of antemortem compression.

## Introduction

Assessing skin lesions that show a patterned shape of injury is vital in forensic pathology. Ligature mark is a well-known compression marker on the neck of hanging or strangulation cases. Analyzing the patterned grooves in the neck found at autopsy can help determine the type of hanging or strangulation^1–3^. The visibility of neck ligature marks is highly dependent on the nature and texture of the ligature^4^. Soft or hard ligature marks are placed on the neck using a variety of tools, including electrical cords, telephone cords, wires, ropes, belts, and ties^4–6^. If the ligature is made of a soft material, such as a towel, the grooves may be faint, barely visible, and not very sharp^4–6^. Existing forensic literature describes various biomarkers and methods for the differential diagnosis of vital and postmortem wounds; however, their effectiveness has yet to be fully elucidated.

The determination of bioreactivity in human skin wounds remains a challenge for forensic pathologists. The complex physiological phenomena that occur at the local and systemic levels when an external force or noxa comes into contact with a living body are called “vital reactions” in forensic medicine^4^. Gross and microscopic responses of cadaveric tissues can be useful in distinguishing between ante- and post-mortem injuries and in determining whether the injuries persist for life. The extravasation of red blood cells and hemoglobin into the soft tissues surrounding the wound is often regarded as a vital sign of local response, and caution should be exercised when interpreting such findings alone as a definitive vital sign^7,8^. It is accompanied by systemic biological reactions such as cyanosis, edema, and congestion, and in some cases, especially in severely degraded or exhumed remains, even gross hemorrhage may not be visible^9^.

In many cases, a single vitality marker is of limited value because it is partially explained by resuscitation attempts or events that occur during the supra-vital period^10^. The symptoms of a localized vital response at the site of injury such as the initiation of inflammatory processes with polymorphonuclear leukocyte migration require more time than is typically observed in traumatic deaths such as hanging or ligature strangulation^10^. In these cases, survival is often very short, mostly within minutes^11^; this is not enough time to generate vital signs such as the initiation of the wound healing process with a general inflammatory response^12^. Several researchers have recommended immunohistochemical analyses as valuable tools in forensic studies^13,14^. However, few validated immunohistochemical markers, such as ligature marks, can aid in solving skin wound vitality and age estimation issues^4–6,15^.

The most ubiquitous system of protection is a group of genes encoding heat shock proteins (HSPs)^16^. HSPs belong to a large family of molecular chaperones classified by their molecular weight, including HSP27, HSP40, HSP60, HSP70, and HSP90^17^. These proteins provide some degree of cellular resistance to hyperthermia, hypoxia, oxidative stress, various types of toxins, radiation, and more^18^. HSPs are intimately involved in several processes, including protein folding, protein transport, and protein complex assembly/degradation^16^. Therefore, these proteins are thought to play a central role in proteostasis. Some HSPs promote the folding of newly created proteins, whereas others are induced during physiological stress and manage the extra burden of stress-induced misfolding of proteins. Additionally, HSPs are involved in determining the fate of misfolded proteins by either refolding them or directing them to the ubiquitin degradation pathway for degradation^19^. These functions of HSPs imply changes in HSP expression at the wound site.

In the present study, we investigated the immunohistochemical expression of HSP27 and HSP70 in neck skin specimens obtained from forensic autopsy cases and discussed their potential as markers for the diagnosis of compression.

## Results

### Expression of HSP27 and 70 in neck compression cases

**Figure 1:**
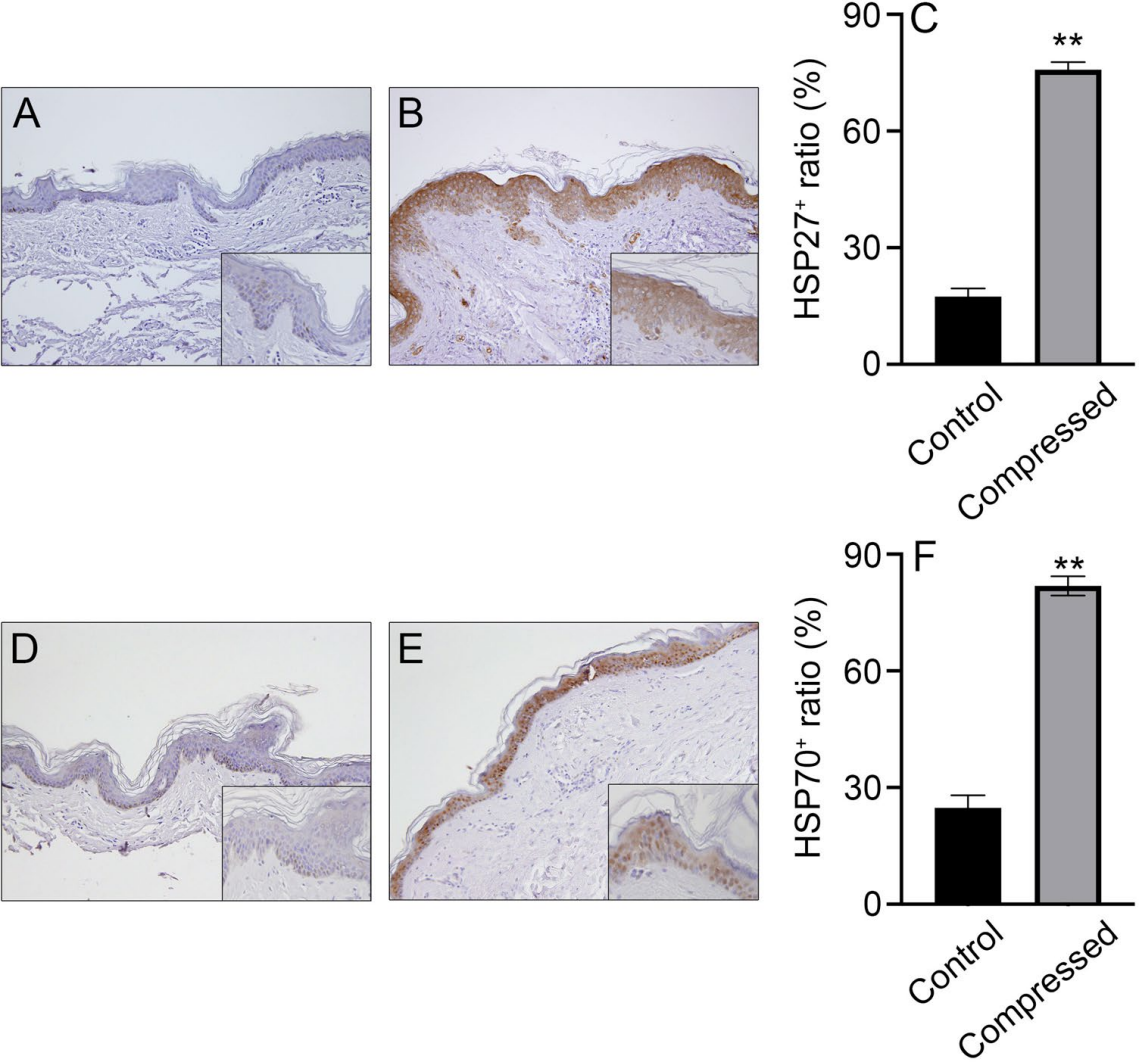
Immunohistochemical analysis. Immunohistochemical analysis were performed by using anti-HSP27 (**A**,**B**) or anti-HSP70 (**D**,**E**) mAbs in the human skin samples. (**A**,**D**) Control; (**B**,**E**) compressed neck skin. Original magnification, × 200; inset, × 400. (**C**,**F**) The ratio of HSP27 positives (**C**) and HSP70 positives (**F**) in the corresponding keratinocytes in the skin sample. **P < 0.01.

**Figure 2:**
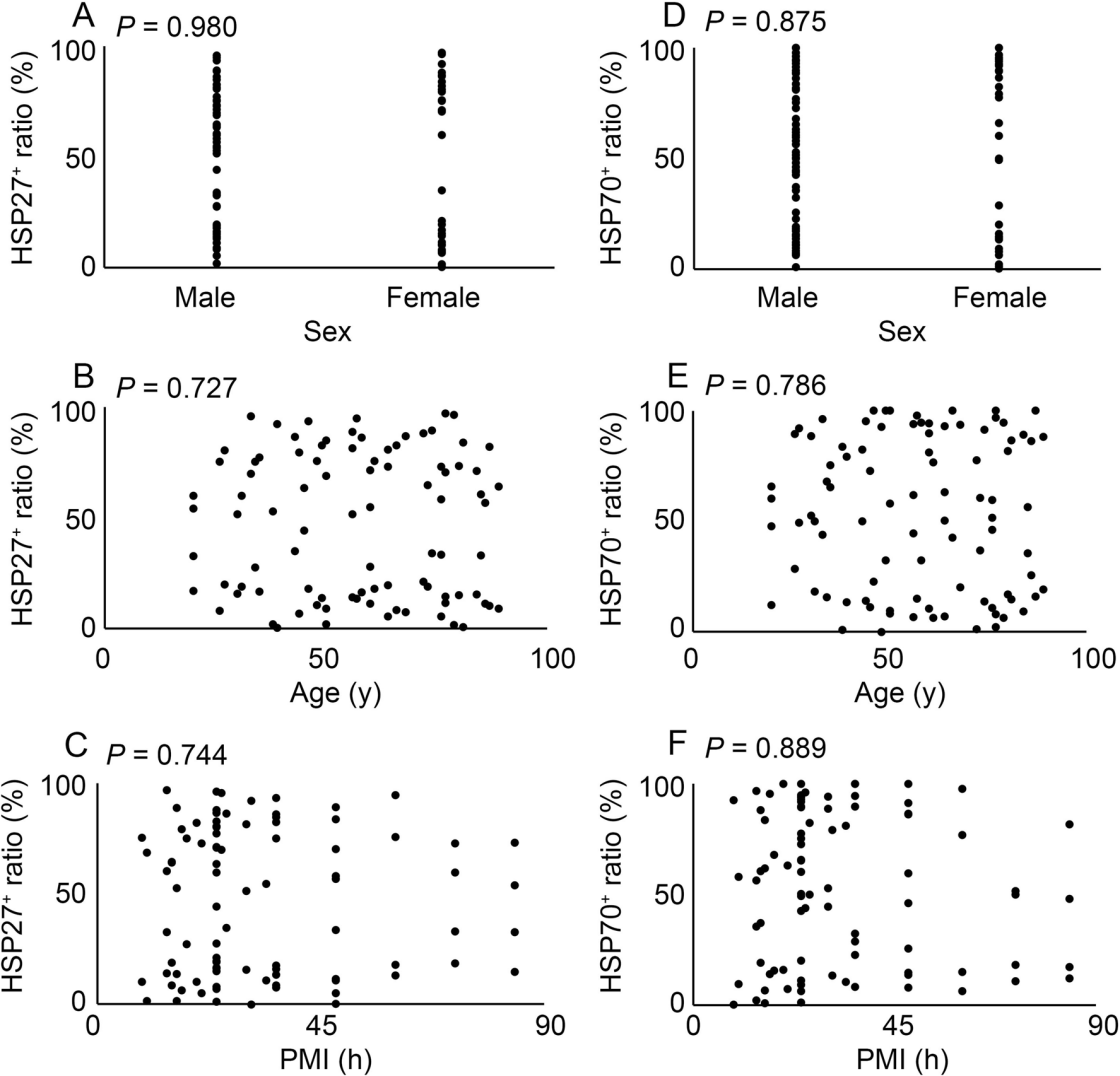
The relation between sex (**A**,**D**), age (**B**,**E**) or postmortem intervals (PMI) (**C**,**F**) and HSP27 expression (**A**–**C**) or HSP70 expression (**D**–**F**) in all cases. These results were obtained with Spearman’s correlation coefficient by rank test.

We investigated the distribution of HSP27 and HSP70 in the compressed neck skin samples. Both HSP27 and HSP70 were not detected in most of intact skin samples (Fig. 1A,D, and Supplemental Fig. S1). Positive signals for HSP27 and HSP70 were observed predominantly in keratinocytes for most ligature marks, respectively (Fig. 1B,E, and Supplemental Fig. S1). Subsequently, we performed semi-quantitative analyses of the immunohistochemical images in order to evaluate the forensic significances of HSP27 and HSP70 in neck compression cases. The ratio of HSP27 to HSP70 expression in keratinocytes was significantly enhanced in the compressed skin sample compared to that in the control samples (Fig. 1C,F). Furthermore, our evaluation of the influence of several factors, such as sex, age, and post-mortem interval (PMI), on the expression of HSP27 and HSP70 in the neck skin indicated that none of the three factors had a significant influence on HSP27 and HSP70 expression in the neck skin (Fig. 2). These observations imply that the expression of HSP27 and HSP70 is useful for determining antemortem neck compression.

### Influence of ligature type and compression method on the expression of HSP27 and HSP70 in the neck skin

**Figure 3:**
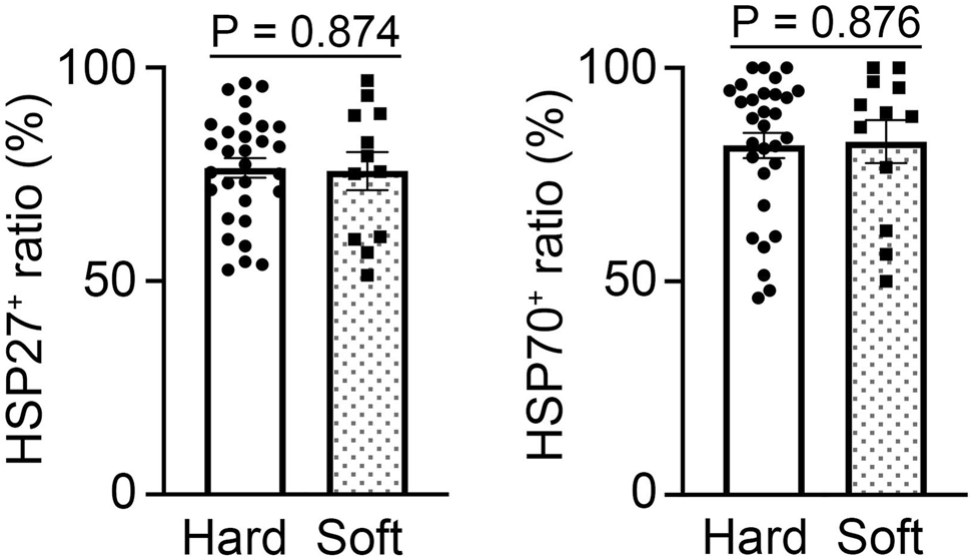
The frequency of appearance of HSP27 and 70 in hard and soft ligatures. Hard, n = 30; soft, n = 12. The ratio of HSP27 positives and HSP70 positives in the corresponding keratinocytes in the skin sample.

In forensic practices, different types of ligatures such are used for the neck compressions. To evaluate the influence of ligature type on the expression of HSP27 and HSP70 expression, we grouped the skin samples into hard and soft ligature mark groups (Fig. 3; hard, n = 30; soft, n = 12), however, no significant differences in the expression of HSP27 and HSP70 were observed between the hard and soft groups.

## Discussion

Estimating the age and vitality of a ligature mark is key to distinguishing between pre- and post-mortem wounds, to distinguish between suicide or homicide, and not missing a crime. In forensic pathology, the evaluation of ligature vitality and age remains a hurdle to overcome^10^. Neck compression can make it difficult to determine the cause of death if there are faint or absent ligature marks on the neck or few internal findings. We previously examined the immunohistochemical expression of ubiquitin in neck skin samples to verify its forensic utility in determining wound vitality^5^. Ubiquitin expression was lower in neck compression samples than in intact skin samples. This ubiquitin depletion of compressed skin is possible with the necessary forensic precision because it is caused by the established ubiquitin retention mechanism, conversion of ubiquitin from conjugated to free forms, and failure to synthesize ubiquitin de novo. However, relying on a singular marker is insufficient from a forensic safety perspective and warrants the inclusion of multiple markers for making accurate diagnosis.

The microscopic manifestation of inflammation in hanging skin samples has been confirmed in several studies. The analyzed molecules with satisfactory results were obtained for tryptase, IL-1β, IL-15, CD1a, CD15, MHC-II, fibronectin, cathepsin D, P-selectin, and aquaporin (AQP)3^4,6^. AQP, a membrane channel protein, is involved in water transport across cell membranes^20^. Among AQPs, AQP3 appears to be a reliable marker of neck compression^6^. In addition, the distribution of MHC class II cells, mast cells, and Langerhans cells at the ligature mark has been investigated immunohistochemically^9,21^. Langerhans cells play important roles in the initial response to tissue damage. The results showed that CD1a^+^ dendritic cells were predominant in the ligature marks and iNOS was overexpressed. Legaz et al. immunohistochemically showed a very strong positive reaction of fibronectin in the dermis of the ligature mark, an increased characteristic granule pattern staining of cathepsin D in the basal layer of the epidermis, and weak positive immunoreactivity of P-selectin^7,22^.

Turillazzi et al. studied the expression of TNF-α, IL-10, CCL2, CD3, CD4, CD8, CD20, CD45, and CD68 however, no significant results were reported^23^. A strong patchy positivity for extracellular tryptase was observed immunohistochemically in the skin connective tissue taken from the upper and lower margins of the hanging marks^23,24^. High levels of CD15 and tryptase were observed in the superficial epidermis of the hanging marks, but no positive immunohistochemical results were observed in postmortem samples^23^. The expression of CD15 antigen is associated with an active response of IL-15 in the subdermal connective tissue and perivascular space, and this co-expression indicates that IL-15 is an inflammatory cytokine that moves leukocytes from the lumen to the site of injury^24,25^.

HSPs are molecular chaperones that protect and support cells under stress and lethality conditions^26^. The expression of HSPs is promoted by all external and internal physiological stresses, such as energy (ATP) deficiency, oxidative stress, ischemia, and chemotherapeutic agents, as well as hypothermic and hyperthermic effects^26–28^. HSP27 belongs to the family of small HSPs (12–43 kDa). The function of human HSP27 is regulated by stress-induced phosphorylation of Ser-15, Ser-78, and Ser-82^29,30^, and it plays a role in neurodegenerative, atherosclerosis, and other diseases^31,32^. Additionally, HSP27 plays an important role in controlling cancer development, progression, metastasis, drug resistance, and cell apoptosis and may be an indicator of poor disease prognosis^33^. HSP27 overexpression has been reported to correlate with peritoneal metastasis in epithelial ovarian cancer^34^. HSP27 in peripheral blood promotes angiogenesis by increasing VEGF gene transcription and VEGF receptor type 2 activation in breast cancer cells^35^. These findings imply that HSP27 plays an important role in ischemic environments including cancer.

HSP70, the most widely studied HSP of the family, is expressed at low levels in healthy cells under normal environmental conditions. However, under the influence of heat shock, hypoxia, ischemia, radiation, and infection conditions, HSP70 expression is significantly upregulated compared to normal conditions^36,37^. During myocardial ischemia, HSP70 expression is upregulated and promotes myocyte health by inhibiting the remodeling and refolding of misfolded proteins and apoptosis-inducing factor (AIF) activity^38^. Furthermore, HSP70 plays an important role in cancer development, and its abnormally high expression has been found in cancer cells^17,39–42^. Cao et al. have demonstrated that in vitro exposure of neonatal rat pulmonary microvascular endothelial cells (PMVECs) to hypoxia upregulated HSP70 expression levels compared to cells maintained under normal oxygen conditions^43^. In addition, Madaeva et al. demonstrated a direct relationship between oxidative stress levels and HSP70 expression in apneic patients^44^. Hypoxia also induces HSP70 expression in many organs, including the heart, liver, and intestine^45–49^. Compressed neck skin appears to be in more severe focal ischemia with cell damage, and HSP70 expression may be upregulated to protect the skin cells from hypoxic cell damage.

HSP27 and HSP70 have also been reported to play important roles in early corneal wound healing^50,51^. Wounds induce the upregulation of the expression HSPs, particularly in the epidermis^52–54^. Wound sites complicated by ischemia, infection, and related factors are harsh environments for cells involved in the repair process; therefore, inducible HSP70 is abundant and contributes to protein homeostasis and cell survival within healing wounds^55,56^. In addition, Doberentz et al. demonstrated that in the lung tissue in fire-related cases, HSP27 and HSP70 in the bronchi of the lung tissue are rapidly expressed in large amounts within seconds or minutes after exposure to heat stress to protect the cells^57,58^. In addition, immunohistochemical studies of HSP70 can help in diagnosing hypoxic/ischemic brain damage during death^59^. These findings strongly support that short-term and substantial upregulation of both HSP27 and HSP70 expression in neck skin may be a hallmark of compression.

However, there are some challenges; for instance, when wide, soft, or yielding materials are used for hanging, the mark is discontinuous or absent, and is insufficient to reliably distinguish it from postmortem lesions as the reaction progresses^23,60^. We evaluated differences in HSP27 and HSP70 expression using compressed skin samples from hard and soft ligatures. No significant differences were observed between the hard and soft ligature groups. The reliability of immunohistochemistry is influenced by a multitude of factors; therefore, a single immunohistochemical biomarker on its own is of limited value. Therefore, validation is still necessary to promote consistency among immunohistochemical markers and improve their reliability in the routine diagnosis of vital reactions^8^. However, our findings showed that the detection of HSP27 and HSP70 in the skin of the neck is possible with forensic accuracy.

## Methods

### Antibodies

The following monoclonal antibodies (mAbs) were used for immunohistochemical analysis in the present study: mouse anti-human HSP27 (sc-13132, Santa Cruz Biotechnology, Dallas, TX) and mouse anti-human HSP70 (sc-32239, Santa Cruz Biotechnology).

### Compressed neck skin samples

A total of 45 compressed neck skin samples were obtained from the following forensic autopsy cases with a postmortem interval of < 84 h. Out of the 45 samples, they were 42 ligature marks (32 hanging and 10 strangulation cases) and 2 manual strangulation marks. The remaining one is a case of 60 s year-old male whose neck was compressed by the handle of a cultivator. In each case, the cause of death was carefully determined based on autopsy, histopathological findings and toxicological data. Intact skin from the same individual was used as the control. The detailed profiles of all cases (sex, age, and postmortem intervals) are shown in Supplementary Table 1.

### Immunohistochemical analysis

Immunohistochemical analysis was performed as described in a previous study^5^. Briefly, skin specimens were fixed in 4% formaldehyde solution buffered with PBS for 4–7 days, embedded in paraffin, and sectioned at a thickness of 4 µm. Subsequently, deparaffinized sections were incubated with PBS containing 1% normal goat serum and 1% bovine serum albumin (BSA) to reduce nonspecific reactions. Thereafter, the sections were further incubated with anti-HSP27 or anti-HSP70 mAbs (2 μg/ml in each antibody) for 12–17 h at 4 °C. After the incubation with biotinylated secondary antibodies, immune complexes were visualized using Catalyzed Signal Amplification System (Dako, Kyoto, Japan) according to the manufacturer’s instructions. As a negative control, sections were incubated with non-immunized and isotype-matched mouse IgG (sc-2025, Santa Cruz Biotechnology) instead of the primary antibodies and no positive signal was found, indicating the specificity of the antibodies.

### Morphometrical analysis

Semiquantitative analyses on immunohistochemical images of HSP27^+^ and HSP70^+^ keratinocytes in the compressed neck skin and control samples were performed. In order to evaluate HSP27 and HSP70 expression in the skin samples, 5 high-power fields (× 400) were randomly selected, and the ratios of HSP27^+^ or HSP70^+^ keratinocytes to the total number of corresponding keratinocytes were calculated as described previously^5,6^. The average values of the 5 selected fields were evaluated as an indicator for HSP27 and HSP70 expression in each case. Morphometric evaluation was blindly performed by two investigators (S.Z and Y.I) without the prior knowledge of the samples.

### Statistical analysis

The mean and standard error of the means (SEM) were calculated. Statistical analysis was performed using analysis of variance or Mann–Whitney *U*-test. Statistical significance was set at *P* < 0.05. Correlation analysis was performed using the nonparametric Spearman’s correlation coefficient. Statistical significance was set at* P* < 0.05.

### Ethical approval

This study was approved by the Research Ethics Committee of Wakayama Medical University (No. 3313). All the procedures were performed in accordance with the Declaration of Helsinki Principles. Moreover, this study was conducted using autopsy records from the past, and we could not obtain informed consent from the bereaved family for the use of these records. Therefore, we conducted this study in accordance with the "Ethical Guidelines for Medical Research Involving Human Subjects (enacted by the Ministry of Health, Labor, and Welfare in Japan), Section 12–1 (2) (a) (c).” This was a de-identified study using archived tissue obtained from judicial autopsy cases, and the information on the implementation of the study was posted on our website (https://www.wakayama-med.ac.jp/dept/igakubu/160420/index.html). If there was a request to refuse the use of the samples for research, they were excluded from samples of this study. In addition, the review board of the Research Ethics Committee of Wakayama Medical University waived the need for written informed consent from the relatives of the individuals studied in accordance with the national legislation and the institutional requirements (No. 3313).

## Supplementary Information

Supplementary Information.

## Data Availability

The authors declare that all data are available in the article file or available from the corresponding authors, Yuko Ishida and Toshikazu Kondo, upon reasonable request.
