# Supplementary material for: Forensic application of epidermal expression of HSP27 and HSP70 for the determination of wound vitality in human compressed neck skin
Source: Sci Rep. 2023 Apr 24;13:6692. doi: 10.1038/s41598-023-33799-4 (PMC10126125; doi:10.1038/s41598-023-33799-4)
Supplement: Supplementary file 1 — Supplementary Information. [file 41598_2023_33799_MOESM1_ESM.docx]

**Supplemental table 1.** Cases profile

| Number | Male/Female | Age (y) | | Postmortem interval (h) | |
| --- | --- | --- | --- | --- | --- |
|  |  | Range | Mean | Range | Mean |
| 45 | 30/15 | 20-89 | 57.2 | 9-84 | 32.8 |


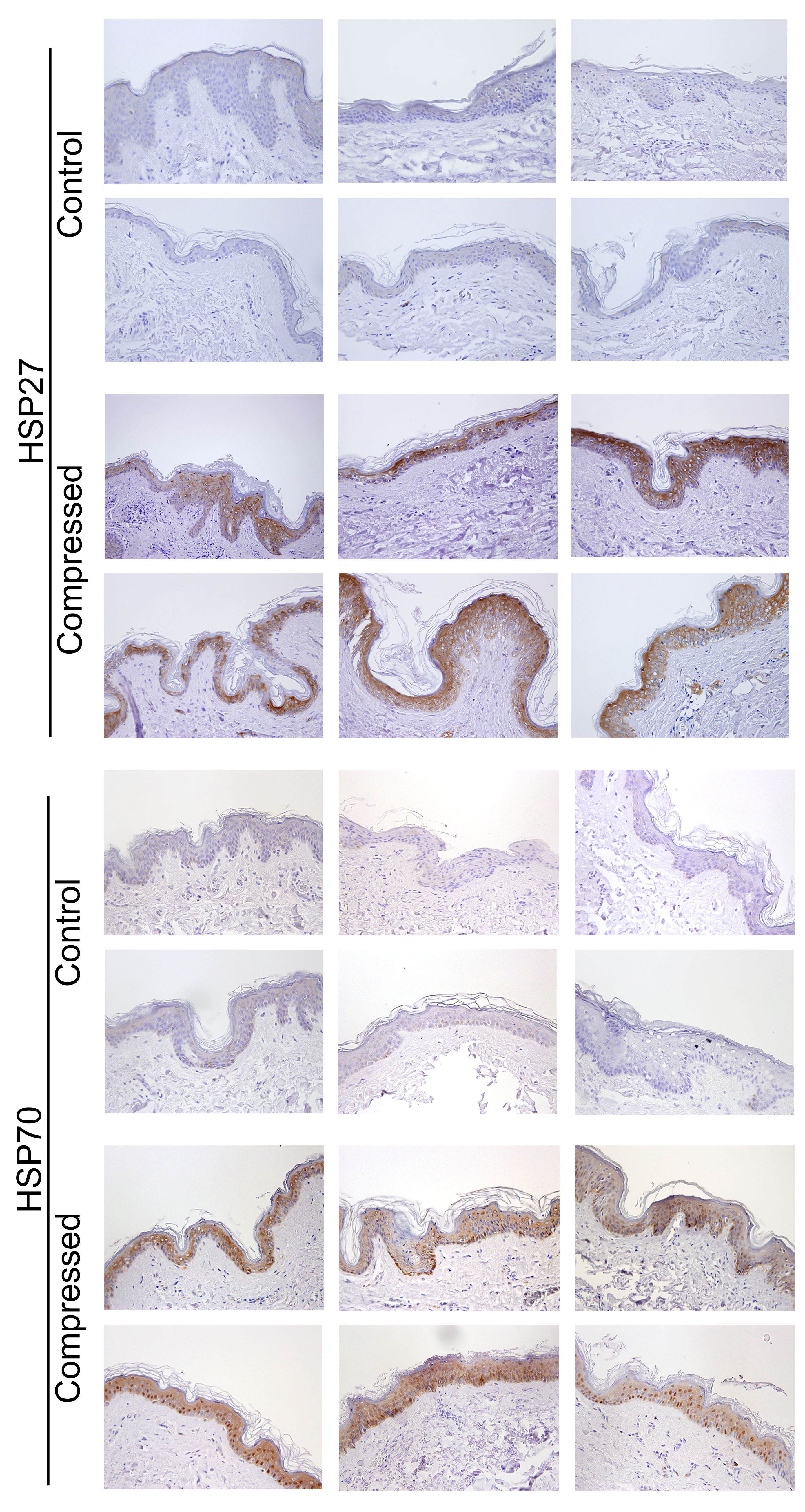


**Supplemental figure 1.** Immunohistochemical analysis. Immunohistochemical analysis were performed by using anti-HSP27 or anti-HSP70 mAbs in the human skin samples. Some results of immunohistochemical analysis are shown. Original magnification, × 200.
